# Supplementary material for: Voluntary Surveillance Program for Equine Influenza Virus in the United States during 2008–2021
Source: Pathogens. 2023 Jan 27;12(2):192. doi: 10.3390/pathogens12020192 (PMC9961984; doi:10.3390/pathogens12020192)
Supplement: Supplementary file 1 [file pathogens-12-00192-s001.zip › pathogens-2116819-File S1.pdf]

INFORMATION ONLY

Equine Respiratory Pathogens Surveillance Questionnaire

Date: ___________________ ***THIS FORM MUST ACCOMPANY ALL SAMPLES****

Designated Contact: _____________________________________ Phone (DC): ______________________

Veterinarian / Clinic: ____

Address: ____

Email: ____

Phone (clinic): __________________________________ Fax (clinic): _______________________________

Owner: ____ Phone: ____________________________________

Horse ID: ____ Age: _______ Sex: ______ Breed: ______

Occupation (circle one): Racing Show Pleasure Breeding Other __________________________________

Sample submitted (circle): blood nasal swab other ____

Vaccination history (if known):

Disease	Vaccine product last used	Date vaccinated or time since last dose	Number of doses given per year
EHV1&4			
Influenza			
S. equi			

How many days has the horse been showing signs prior to sampling? ______________________________

Has the horse been transported during the past 14 days? _______________________________________

How many other horses on the premises are showing signs? _____________________________________

CLINICAL SIGNS AND SEVERITY AT THE TIME OF TESTING				
**Nasal discharge**	None observed	Mild	Moderate	Severe
	If present:	Serous	Mucoid	Mucopurulent
**Cough**	None	Occasional	Intermittent	Frequent
**Depression; lethargy**	None observed	Mild; easily roused	Moderate; reluctant to move voluntarily	Severe; unwilling to move
**CNS signs**	**Describe if present:**			
**Fever (°F)**	**Highest temperature recorded on day of testing:**			
**Limb swelling**	None observed	Mild, tendons visible	Moderate, tendons not visible	Severe; lame
**Loss of appetite**	None observed	Slight	Moderate	Complete anorexia
**Ocular discharge**	None observed	Mild	Moderate	Severe
	If present:	Serous	Mucoid	Mucopurulent

Please make any additional comments on the back of this form.

The Real-Time PCR Research & Diagnostics Core Facility at Davis
Attn: Dr. Nicola Pusterla
Department of Medicine and Epidemiology
School of Veterinary Medicine
University of California

1275 Med Sci Drive
One Shields Avenue
Davis, CA 95616
Phone: 530-752-1039
Fax: 530-754-6862

****Please direct questions to Equine Technical Services 1-866-349-3497 or USEquinePV@merck.com****
